# Supplementary material for: The Development of Thematic Core Collections in Cassava Based on Yield, Disease Resistance, and Root Quality Traits
Source: Plants (Basel). 2023 Oct 4;12(19):3474. doi: 10.3390/plants12193474 (PMC10574880; doi:10.3390/plants12193474)
Supplement: Supplementary file 1 [file plants-12-03474-s001.zip › Santos et al_Plants_2023 Table S1.pdf]

Supplementary material

Table S1. Pairs of cassava genotypes with relatedness >0.95 obtained through identity by state (IBS) using 20,023 SNPs on 172 cassava individuals selected from different thematic collections (CC_Root_quality - root quality and CC_Yield - for yield traits).

1- BGM-0082/ BGM-0091	37- BGM-0817/ BGM-1423	73- BGM-0250/ BGM-1679
2- BGM-0123/ BGM-0248	38- BGM-0341/ BGM-1429	74- BGM-1227/ BGM-1679
3- BGM-0123 /BGM-0250	39- BGM-0817/ BGM-1429	75- BGM-1569/ BGM-1679
4- BGM-0248/ BGM-0250	40- BGM-1423/ BGM-1429	76- BGM-0145/ BGM-1716
5- BGM-0213/ BGM-0286	41- BGM-0307/ BGM-1465	77- BGM-0145/ BGM-1721
6- BGM-0287/ BGM-0303	42- BGM-0341/ BGM-1474	78- BGM-1716/ BGM-1721
7- BGM-0215/ BGM-0316	43- BGM-0817/ BGM-1474	79- BGM-0368/ BGM-1817
8- BGM-0093/ BGM-0320	44- BGM-1423/ BGM-1474	80- BGM-0882/ BGM-1817
9- BGM-0287/ BGM-0389	45- BGM-1429/ BGM-1474	81 - BGM-0123/ BGM-1834
10- BGM-0303/ BGM-0389	46- BGM-1359/ BGM-1489	82- BGM-0248/ BGM-1834
11- BGM-0287/ BGM-0442	47- BGM-1361/ BGM-1489	83- BGM-0250/ BGM-1834
12- BGM-0303/ BGM-0442	48- BGM-1371/ BGM-1489	84- BGM-1227/ BGM-1834
13- BGM-0389/ BGM-0442	49- BGM-0579/ BGM-1498	85- BGM-1569/ BGM-1834
14- BGM-0527/ BGM-0529	50- BGM-1496/ BGM-1502-M	86- BGM-1679/ BGM-1834
15- BGM-0270/ BGM-0591	51- BGM-0341/ BGM-1507	87- BGM-0341/ BGM-1880
16- BGM-0384/ BGM-0601	52- BGM-0817/ BGM-1507	88- BGM-0817/ BGM-1880
17- BGM-0408/ BGM-0694	53- BGM-1423/ BGM-1507	89- BGM-1423/ BGM-1880
18- BGM-0341/ BGM-0817	54- BGM-1429/ BGM-1507	90- BGM-1429/ BGM-1880
19- BGM-0368/ BGM-0882	55- BGM-1474/ BGM-1507	91- BGM-1474/ BGM-1880
20- BGM-0212/ BGM-1034	56- BGM-0123/ BGM-1569	92- BGM-1507/ BGM-1880
21- BGM-0666/ BGM-1064	57- BGM-0248/ BGM-1569	93- BGM-0093/ BGM-2234
22- BGM-0533/ BGM-1130	58- BGM-0250/ BGM-1569	94- BGM-0320/ BGM-2234
23- BGM-0123/ BGM-1227	59- BGM-1227/ BGM-1569	95- BGM-1626/ BGM-2234
24- BGM-0248/ BGM-1227	60- BGM-1078/ BGM -1624	96- BGM-0341/ BGM-2245
25- BGM-0250/ BGM-1227	61- BGM-0093/ BGM-1626	97- BGM-0817/ BGM-2245
26- BGM-0287/ BGM-1269	62- BGM-0320/ BGM-1626	98- BGM-1423/ BGM-2245
27- BGM-0303/ BGM-1269	63- BGM-0666/ BGM-1638	99- BGM-1429/ BGM-2245
28- BGM-0389/ BGM-1269	64- BGM-1064/ BGM-1638	100- BGM-1474/ BGM-2245
29- BGM-0442/ BGM-1269	65- BGM-0189/ BGM-1643	101- BGM-1507/ BGM-2245
30- BGM-0212/ BGM-1355	66- BGM-0189/ BGM-1671	102- BGM-1880/ BGM-2245
31- BGM-1034/ BGM-1355	67- BGM-1643/ BGM-1671	103- BGM-1311/ BGM-2275
32- BGM-1359/ BGM-1361	68- BGM-0189/ BGM-1677	104- BGM-2095/ BGM-2339
33- BGM-1359/ BGM-1371	69- BGM-1643/ BGM-1677	105- BGM-1249/ BGM-2355
34- BGM-1361/ BGM-1371	70- BGM-1671/ BGM-1677	106- BGM-2249/ BGM-2356
35- BGM-0443/ BGM-1395	71- BGM-0123/ BGM-1679	107- BGM-0579/ BRS-Amansa-Burro
36- BGM-0341/ BGM-1423	72- BGM-0248/ BGM-1679	108- BGM-1498/ BRS-Amansa-Burro
