# Supplementary material for: The Development of Thematic Core Collections in Cassava Based on Yield, Disease Resistance, and Root Quality Traits
Source: Plants (Basel). 2023 Oct 4;12(19):3474. doi: 10.3390/plants12193474 (PMC10574880; doi:10.3390/plants12193474)
Supplement: Supplementary file 1 [file plants-12-03474-s001.zip › Santos et al_Plants_2023 Table S2.pdf]

Supplementary material

Table S2. The broad-sense heritability (h^2) of the quantitative traits used in the establishment of thematic collections

N.º	Trait	Abbreviation	h^2
1.	Plant height	Pl.H	0.503
2.	Plant type	Pl.T	0.415
3.	Shoot yield	ShY	0.407
4.	Marketable fresh root yield	C.FRY	0.230
5.	Unmarketable fresh root yield	NC.FRY	0.147
6.	Total fresh root yield	T.FRY	0.383
7.	Dry matter content	DMC	0.395
8.	Dry root yield	DRY	0.345
9.	Harvest index	HI	0.343
10.	Leaf retention	Pl.LR	0.213
11.	Plant vigor at 1.5 months	Pl.V1.5M	0.230
12.	Plant vigor at 12 months	Pl.V12M	0.600
13.	Average number of roots per plant	Ro.NP	0.147
14.	Mite severity	P.MS	0.466
15.	Bacterial blight severity	D.CBB	0.157
16.	Rust severity	D.RS	0.279
17.	Frogskin severity	D.FS	0.099
18.	Hydrogen cyanide content	Ro.HCN	0.916
19.	Total carotenoid content	Ro.TCC	0.953
