# Supplementary material for: The Development of Thematic Core Collections in Cassava Based on Yield, Disease Resistance, and Root Quality Traits
Source: Plants (Basel). 2023 Oct 4;12(19):3474. doi: 10.3390/plants12193474 (PMC10574880; doi:10.3390/plants12193474)
Supplement: Supplementary file 1 [file plants-12-03474-s001.zip › Santos et al_Plants_2023 Table S3.pdf]

Supplementary material

Table S3. Geographical location and characterization of the cassava field trials.

Year	Experimental Design[1]	Location	City	Altitude (m)	Coordenates	Soil type[2]
2011	RCBD	Embrapa	Cruz das Almas	215	12°40'36.7"S, 39°05'08.0"W	YL
	RCBD	Embrapa	Cruz das Almas	210	12°40'32.6"S, 39°05'13.7"W	YL
2012	ABD	Embrapa	Cruz das Almas	215	12°40'36.7"S, 39°05'08.0"W	YL
	ABD	Embrapa	Cruz das Almas	210	12°40'32.6"S, 39°05'13.7"W	YL
	ABD	Embrapa	Cruz das Almas	200	12°40'47.4"S, 39°05'00.2"W	YL
2013	RCBD	Coopamido	Laje	180	13°06'38.4"S, 39°16'20.4"W	RL
	RCBD	UFRB	Cruz das Almas	210	12°39'25.9"S, 39°04'58.8"W	YL
	RCBD	Embrapa	Cruz das Almas	210	12°40'32.6"S, 39°05'13.7"W	YL
2014	RCBD	Embrapa	Cruz das Almas	217	12°40'22.8"S, 39°05'06.1"W	YL
	ABD	Embrapa	Cruz das Almas	216	12°40'22.8"S, 39°05'01.5"W	YL
	ABD	Embrapa	Cruz das Almas	216	12°40'22.8"S, 39°05'01.5"W	YL
	ABD	Coopamido	Laje	180	13°06'39.6"S, 39°16'17.6"W	YL
	ABD	Coopamido	Laje	175	13°06'35.6"S, 39°16'19.3"W	YL
	ABD	Embrapa	Cruz das Almas	216	12°40'22.8"S, 39°05'01.5"W	YL
2015	RCBD	Coopamido	Laje	180	13°06'39.6"S, 39°16'17.6"W	YL
	ABD	Embrapa	Cruz das Almas	216	12°40'22.8"S, 39°05'01.5"W	YL
	ABD	Bahiamido	Laje	196	13°06'38.5"S, 39°16'49.0"W	YL
	ABD	Bahiamido	Valença	40	13°15'33.5"S, 39°14'12.8"W	YRL
	ABD	UFRB	Cruz das Almas	210	12°39'16.4"S, 39°04'53.4"W	YL
	ABD	Bahiamido	Laje	296	13°06'38.5"S, 39°16'49.0"W	YL
2016	ABD	Embrapa	Cruz das Almas	216	12°40'19.5"S, 39°05'02.5"W	YL
	ABD	Embrapa	Cruz das Almas	216	12°40'19.5"S, 39°05'02.5"W	YL
	ABD	Embrapa	Cruz das Almas	216	12°40'19.5"S, 39°05'02.5"W	YL
	ABD	Embrapa	Cruz das Almas	216	12°40'19.5"S, 39°05'02.5"W	YL
2017	ABD	Embrapa	Cruz das Almas	216	12°40'19.5"S, 39°05'02.5"W	YL
2018	RCBD	UFRB	Cruz das Almas	223	12°39'51.4"S, 39°04'15.7"W	YL
2019	RCBD	UFRB	Cruz das Almas	223	12°39'43.5"S, 39°04'12.0"W	YL
	RCBD	UFRB	Cruz das Almas	223	12°39'43.5"S, 39°04'12.0"W	YL
	RCBD	UFRB	Cruz das Almas	223	12°39'43.5"S, 39°04'12.0"W	YL
2020	RCBD	UFRB	Cruz das Almas	223	12°39'49.2"S, 39°03'58.1"W	YL
	RCBD	UFRB	Cruz das Almas	225	12°39'49.2"S, 39°03'58.1"W	YL
2021	ABD	UFRB	Cruz das Almas	225	12°39'49.2"S, 39°03'58.1"W	YL

[1]RCBD: randomized complete block design, ABD: augmented block design; [2]LA - yellow latosol; LV - red latosol; LVA - yellow red latosol.
